# Examining the Interactome of Huperzine A by Magnetic Biopanning

**DOI:** 10.1371/journal.pone.0037098

**Published:** 2012-05-16

**Authors:** Wei Guo, Shupeng Liu, Jinliang Peng, Xiaohui Wei, Ye Sun, Yangsheng Qiu, Guangwei Gao, Peng Wang, Yuhong Xu

**Affiliations:** 1 School of Pharmacy, Shanghai Jiao Tong University, Shanghai, People's Republic of China; 2 Institute of Biomedical Engineering, Shanghai University, Shanghai, People's Republic of China; 3 Med-X Research Institute, Shanghai Jiao Tong University, Shanghai, People's Republic of China; Institute Biomedical Research August Pi Sunyer (IDIBAPS) - Hospital Clinic of Barcelona, Spain

## Abstract

Huperzine A is a bioactive compound derived from traditional Chinese medicine plant Qian Ceng Ta (Huperzia serrata), and was found to have multiple neuroprotective effects. In addition to being a potent acetylcholinesterase inhibitor, it was thought to act through other mechanisms such as antioxidation, antiapoptosis, etc. However, the molecular targets involved with these mechanisms were not identified. In this study, we attempted to exam the interactome of Huperzine A using a cDNA phage display library and also mammalian brain tissue extracts. The drugs were chemically linked on the surface of magnetic particles and the interactive phages or proteins were collected and analyzed. Among the various cDNA expressing phages selected, one was identified to encode the mitochondria NADH dehydrogenase subunit 1. Specific bindings between the drug and the target phages and target proteins were confirmed. Another enriched phage clone was identified as mitochondria ATP synthase, which was also panned out from the proteome of mouse brain tissue lysate. These data indicated the possible involvement of mitochondrial respiratory chain matrix enzymes in Huperzine A's pharmacological effects. Such involvement had been suggested by previous studies based on enzyme activity changes. Our data supported the new mechanism. Overall we demonstrated the feasibility of using magnetic biopanning as a simple and viable method for investigating the complex molecular mechanisms of bioactive molecules.

## Introduction

Huperzine A (Hup A) is a compound found in the traditional Chinese medicine plant Qian Ceng Ta (Huperzia serrata) and has been shown to have neuroprotective effects [1], [2] in Alzheimer disease (AD) patients. It is a potent inhibitor of acetylcholinesterase(AChE) [3]. However, many recent studies have also suggested that it may have other mechanisms including cell protection against apoptosis through reversing the down-regulation of the expression of Bcl-2 and up-regulation of the expressions of Bax and P53 [4], [5], [6], mitochondria protection against dysfunction by preserving major mitochondria enzymes activity and reducing reactive oxygen species (ROS) production [7], interfering with amyloid precursor protein (APP) cleavage [8], [9], etc [10]. However, the detailed molecular mechanisms of most of these pharmacology effects were still not clear. It is generally accepted that Hup A has multiple targets. In order to identify the potential target molecules involved in these effects, Lun Yang and his coworkers published an interesting study of virtual chemical-protein interactome (CPI) analysis, in which they evaluated the possible interactions between Hup A and 401 human protein pockets using the DOCK program [11]. Besides the only validated target AChE, several other putative targets were indicated to suggest some “behind-the-scenes” therapeutic mechanisms of Hup A.

But for systematic evaluation of all the drug's molecular interactions with protein targets in vivo, it remains as a formidable challenge with limited success. In general, there are two approaches: the phenotype-based target discovery and the affinity-based target identification. DNA microarrays were widely used in phenotype based target discovery approaches [12]. Potential drug interactive targets were implicated based on gene expression changes after compound treatment [13], [14]. But genes that were not directly targeted but at upstream or downstream could complicate the interpretation. The affinity based approach is more direct. Potential targets were identified by direct or cooperative binding to the drug itself. For example, Ornithine γ-amino transferase (OAT) was found as the protein target of diazonamide A using a biotinylated form of the drug after affinity purification. This study resulted in the new role of OAT as a chemotherapeutic target [15]. Recently these affinity based approaches were further improved or combined with more sensible analytical methods with more sensitive binding detection and more accurate protein identification [16]. Many new protein targets were uncovered relying on these approaches. Glyoxalase I was identified as a new target of indomethacin by using a polyproline linker to attach small molecule on resins for affinity purification [17]. Such a linker was thought to give the small molecule adequate accessibility to their protein partners. Similarly, the molecular target of resveratrol, a difficult candidate for target identification using conventional affinity strategies was uncovered using the new method drug affinity responsive target stability (DARTS) [18].

In this study, we also took the affinity based approach and developed a magnetic particle mediated screening protocol to maximize the drug-target interaction efficiency. At the same time, the paramagnetic properties of the particles enable convenient and efficient separation of the bound portions from the target mixtures. Therefore, we were able to screen the different proteomes, including a cDNA expression library, and total protein extracts from animal tissues. Although since the detailed screening conditions were different, the outcomes would be different too. Among the wealth of information from different sources, we were able to find certain convergent clues about the molecular mechanism of Hup A.

## Results

### Preparation of Hup A - magnetic particle conjugates (Hup-MPs)

**Figure 1:**
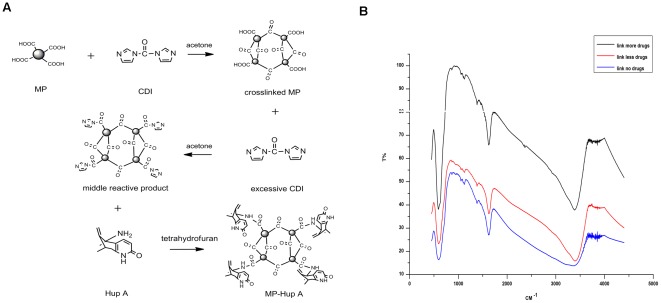
The preparation of Hup-MPs. (a)The reaction scheme of Hup-MPs. (b)FTIR scanning map of MP linked with Hup A(black line: MP linked with more drug; red line: MP linked with a little drug; blue line: MP linked with no drug).

Magnetic nanoparticles were synthesized in the presence of branched polymerized lactic acid as templates as described by Liu,et al [19]. The nanoparticle-polymer matrix was stabilized by CDI crosslinking to yield magnetic particles (MPs). Drugs were conjugated to the MPs via the free carboxyl groups on polymers associated with MPs. Figure 1A showed the reaction scheme. CDI was used as the linker and the resulted linkage should contain an amide bond that can be detected by FTIR. As shown in Figure 1B, the vibration peak at 3340CM^−1^ increased with the amount of Hup A linked and very weak peak was found without the Hup A conjugation. For additional experiments, Drug-biotin-MPs were also made by biotinylated Hup A and ethonalamine (as control) and linked them to streptavidin-MP (Dynabeads M-280 from Invitrogen).

### Hup-MPs mediated interaction and magnetic separation

**Figure 2:**
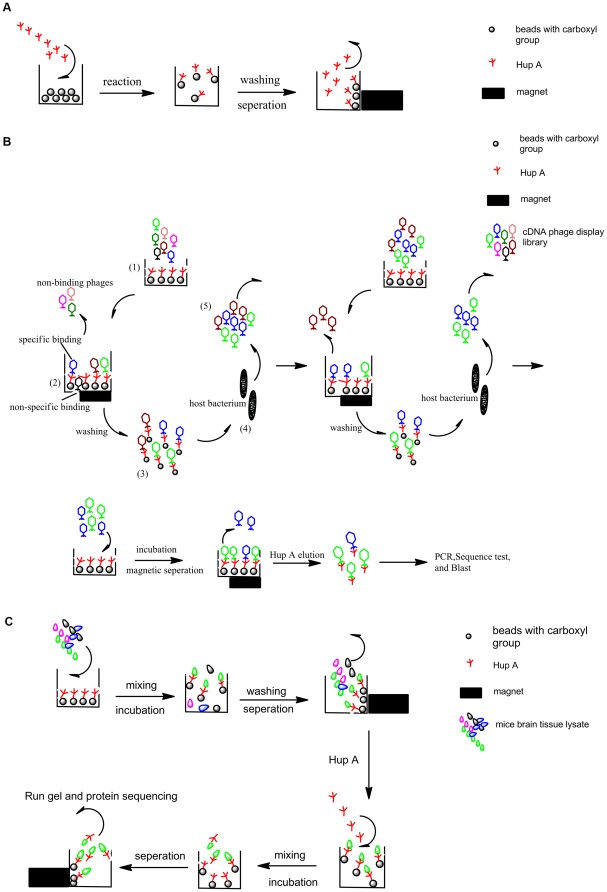
The schematic presentation of MP based strategy for identification of Hup A-target interactions. (a)The attachment of Hup A on the surface of magnetic particles. (b)The strategy of Hup A interacted phages screen from cDNA phage display library. From 1 to 5 was one total round of screening, several rounds of such screen were shown, and the final phages were eluted by Hup A and analyzed. (c)The strategy of Hup A target proteins screen from mice brain tissue lysate. After gel running, the specific protein bands were cut down and identified by MS.

The Hup-MPs could be easily suspended in aqueous solutions, which would allow interaction of the surface Hup A with molecules in the solution. They can also be easily separated from the solution under an external magnetic field, as shown in Figure 2. Based on these schematics, we had used the Hup-MPs for interactions with cDNA phage display libraries (Figure 2B), or brain tissue lysate (Figure 2C). The phages or proteins that were selected were further analyzed based on genomics or proteomics approaches as described below.

### Analysis of cDNA clones in a phage library that interacted with Hup-MPs

Hup-MPs were incubated with a T7 phage cDNA library from human liver cancer cell and bound phages were harvested by magnetic separation and cultured for clonal expansion and next round screening. 5 such consecutive rounds of screening were done and phages collected after the last round of screening and eluted with free Hup A solution were analyzed for their cDNA clones. Parallel experiments were run using non-specific MP controls (MPs without the surface Hup A). No phage clones were selected after the 3rd round of bio-panning. All the procedures above constitute one time screening effort. Table 1 listed all the cDNA sequences found from 6 screening efforts and their putative protein targets. Proteins in italic were highly enriched after each screening effort. Interestingly, 2 of these genes were sequenced repeatedly. The sequences were listed in Table 2. One encodes for part of mitochondrion NADH dehydrogenase subunit 1(MT-ND1) gene, and the other encodes for a section of X chromosome gene.

**Table 1:** Blast result of the gene sequences displayed by all the screened phages.

Homo sapiens isolate cftr13838_B cystic fibrosis transmembrane conductance regulator ATP-binding cassette sub-family C member 7 (CFTR) gene, complete cds; and CTTNBP2 (CTTNBP2) gene, partial cds
Homo sapiens microfibrillar-associated protein 2 (MFAP2), transcript variant 1, mRNA
Homo sapiens fibroblast growth factor 13 (FGF13) gene, complete Cds
Homo sapiens fibronectin1, mRNA (cDNA clone IMAGE:3506187), partial cds
Homo sapiens fibrinogen alpha chain, mRNA (cDNA clone IMAGE:4767540), complete cd
Human heparin cofactor II (HC-II) mRNA, complete cds
Homo sapiens TBXAS1 gene for thromboxane synthase, complete cds
*Homo sapiens ATP synthase, H+ transporting, mitochondrial F1 complex, epsilon subunit, mRNA (cDNA clone MGC:104243 IMAGE:6739745), complete cds(2^nd^ time screening effort)*
The SDHB gene for succinate dehydrogenase complex subunit B iron sulfur (Ip), the PADI2 gene for type II peptidyl arginine deiminase, complete sequence
*Homo sapiens mitochondrion, complete genome(partial sequence of NADH dehydrogenase subunit 1)(4^th^ time screening effort)*
Homo sapiens mitochondrion, complete genome (partial sequence of Cytochrome oxidase subunit I)
Predicted: Homo sapiens similar to ATP-dependent DNA helicase 2 subunit 1 (ATP-dependent DNA helicase II 70 kDa subunit) (Lupus Ku autoantigen protein p70) (Ku70) (70 kDa subunit of Ku antigen) (Thyroid-lupus autoantigen) (TLAA) (CTC box-binding factor 75 kDa subunit)
Homo sapiens ferritin, light polypeptide (FTL), mRNA
Crassostrea gigas tbetaRI gene for TGF-beta Type I receptor, exons 1–10
*Homo sapiens cDNA FLJ39418 fis, clone PLACE6017714, highly similar to prostacyclin receptor(5^th^ time screening effort)*
Homo sapiens ribosomal protein S13, mRNA (cDNA clone MGC:87221 IMAGE:4816284), complete cds
Homo sapiens full-length cDNA clone CS0DA007YC19 of Neuroblastoma
Homo sapiens polycystic kidney disease-associated protein (PKD1) gene, complete cds
*Homo sapiens secreted phosphoprotein 1 (osteopontin, bone sialoprotein I, early T-lymphocyte activation 1) (SPP1), transcript variant 3, mRNA(3^rd^ time screening effort)*
Homo sapiens X-ray repair complementing defective repair in Chinese hamster cells 6 (Ku autoantigen, 70 kDa) (XRCC6), mRNA
Homo sapiens p8 protein (candidate of metastasis 1) (P8), mRNA
Homo sapiens cell growth inhibiting protein 42 mRNA, complete cds
Homo sapiens ERBB receptor feedback inhibitor 1 (ERRFI1), mRNA
Homo sapiens heat shock 70 kDa protein 8, mRNA (cDNA clone MGC:17984 IMAGE:3920744), complete cds
Homo sapiens nucleolar protein 1, 120 kDa, mRNA (cDNA clone MGC:3093 IMAGE:3349415), complete cds

**Table 2:** Gene sequences displayed by the repeatedly screened phages and blast result.

Sequences identified in Hup-MPs bound phage clones	Blast results
AGTTACCCTAGGGATAACAGCGCAATCCTATTCTAGAGTCCATATCAACAATAGGGTTTACGACCTCGATGTTGGATCAGGACATCCCGATGGTGCAGCCGCTATTAAAGGTTCGTTTGTTCAACGATTAAAGTCCTACGTGATCTGAGTTCAGACCGGAGTAATCCAGGTCGGTTTCTATCTACTTCAAATTCCTCCCTGTACGAAAGGACAAGAGAAATAAGGCCTACTTCACAAAGCGCCTTCCCCCGTAAATGATATCATCTCAACTTAGTATTATACCCACACCCACCCAAGAACAGGGTTTGTTAAGATGGCAGAGCCCGGTAATCGCATAAAACTTAAAACTTTACAGTCAGAGGTTCAATTCCTCTTCTTAACAAC*ATACCCATGGCCAACCTCCTACTCCTCATTGTACCCATTCTAATCGCAATGGCATTCCTAATGCTTACCGAACGAAAAATTCTAGGCTATATACAA*	gb|JF682349.1| Homo sapiens mitochondrion, complete genome (gene sequence in italic is the start part of Mitochondrion NADH dehydrogenase subunit 1)
GCTTTGTTCTTTTTTTTTTTTTTTTAGTCTGTTTTCTCTCTTGTTTAGATTGACATAATTCTACTGATTGGTTCACTGACTCTGTCGTCTATCATTTCCACTCTTTTATTGAGCTCATTCAAAGAGTTTTTATTTTAGGTTTTTTTTTTTTTTTT	emb|AL133545.10| Human DNA sequence from clone RP11-386N14 on chromosome X Contains the DUSP21 gene for dual specificity phosphatase 21, the 5′ end of the UTX gene for ubiquitously transcribed tetratricopeptide repeat gene X chromosome and 3 CpG islands, complete sequence

### Capillary electrophoresis confirmation of drug phage interaction

**Figure 3:**
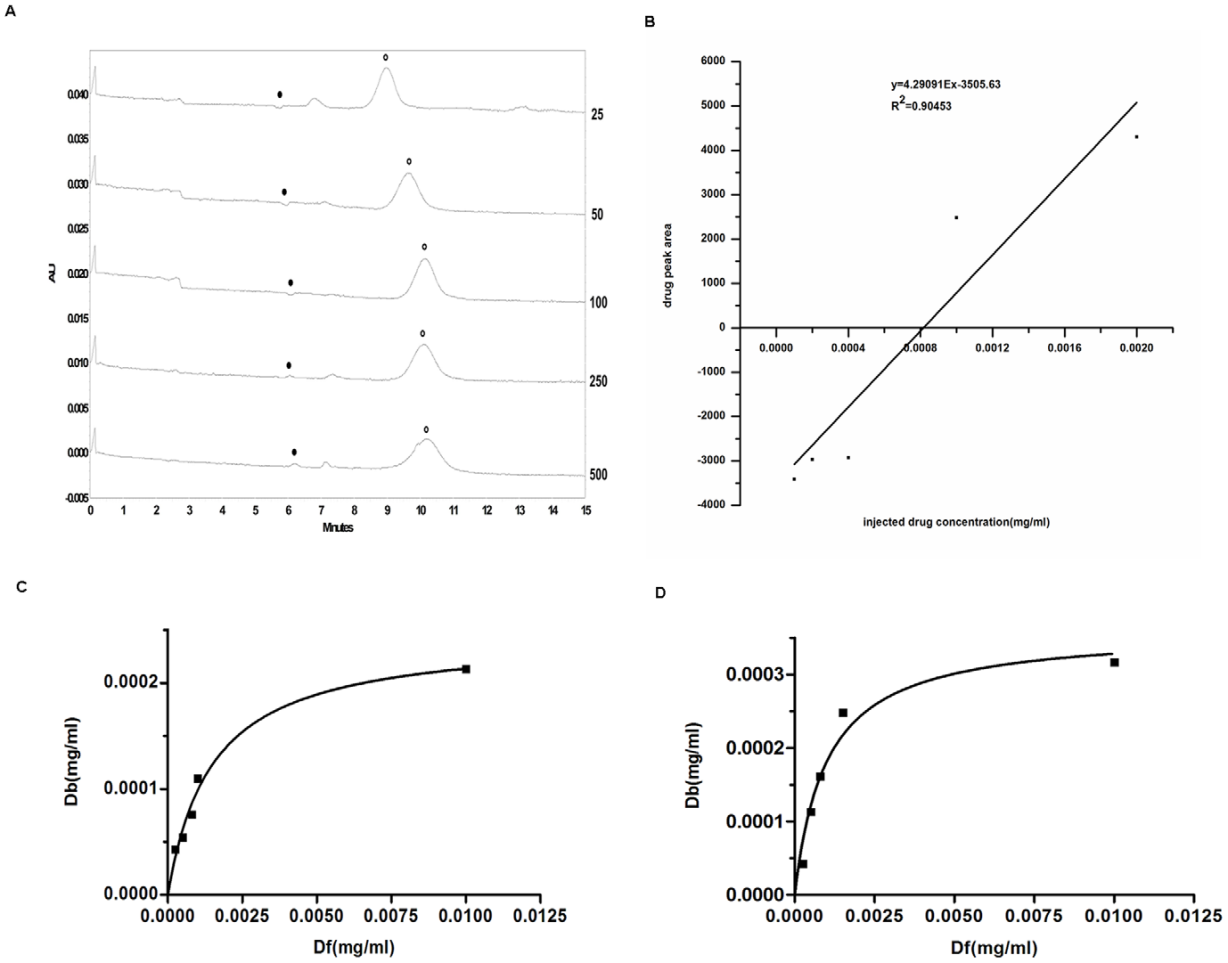
The capillary electrophoresis confirmation on the binding between Hup A and the specific phages. (a)The capillary electrophoresis map of the binding analysis between Hup A and the phage displaying mitochondria gene, drug concentration in the electrophoresis buffer was 0.0008 mg/ml. Hup A concentration injected from top to bottom was 0.0001, 0.0002, 0.0004, 0.001 and 0.002 mg/ml. The corresponding drug/phage ratio was indicated by the number on the right side.(•) Hup A peak; (○) phage peak, including the dissociative phage and drug-bound phage. (b)The curve and trendline between Hup A trough area and concentration using zero interpolation of internal calibration method. (c)The binding curve between Hup A and the specific phage displaying MT-ND1 gene. (d)The binding curve between Hup A and the phage displaying X-chromosome gene.

To exclude the possible interferences from MPs to the interaction between Hup A and selected phages, we used a different analytical method: affinity capillary electrophoresis to characterize the binding. Mixtures of drug and phage at different ratios (the phage numbers were kept constant) were injected and the resulted electrophoregram was shown in Figure 3A. The peak marked by an open circle is the phage peak, and the peak marketed by a filled circle is the unbound Hup A peak, representing the difference between the dissociative drug concentration and drug concentration in the electrophoresis buffer (Df). As drug to phage ratio increased, the size of the unbound drug peak also increased (the unbound drug peak was initially negative because there was a fixed concentration of drug in the buffer). The areas of these unbound drug peaks vs. the amount of drug added were used to fit the Hammer-dreyer plot (Figure 3B). The fitted plot indicated the added drug concentration Dt when the drug peak area was zero, and the bound drug concentration Db was the difference between Dt and Df. The binding constant Kd was obtained by fitting the different Df and Db(Figure 3C and D). The Kd for phage- MT-ND1 was 0.001488 mg/ml and the maximum binding concentration Bmax was 0.0002452 mg/ml (Figure 3C). For the phage-X chromosome gene, the Bmax was 0.0003608 mg/ml and Kd was 0.0009942 mg/ml (Figure 3D). As a control, the binding between Hup A and unrelated phage was also examined by capillary electrophoresis, and no binding could be detected under the same experimental conditions.

### SPR analysis of drug and MT-ND1 protein interaction

**Figure 4:**
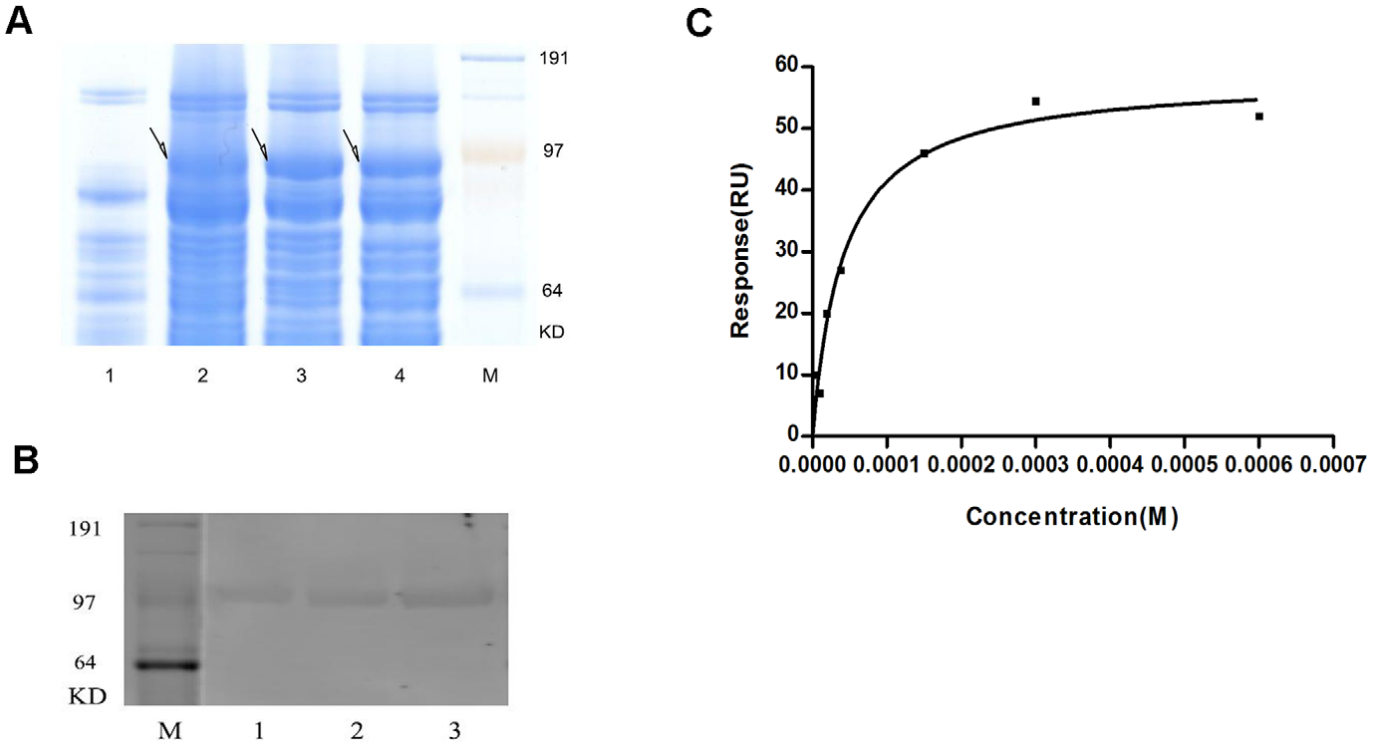
Kinetic analysis of SPR between Hup A and MT-ND1. (a)4–12% SAS-PAGE electrophoresis map of proteins expressed by BL21(DE3) before and after inducement. 1–4 shows the different bands before and after inducement. 1,before inducement; 2,3,4,after inducement. (b)Western blot result of the expressed protein.1–3 shows the bands of loaded proteins with increased volume. C shows the binding curve got from SPR analysis between MT-ND1 and Hup A.

In order to exam the exact binding characteristics between Hup A and the putative protein target, we cloned the MT-ND1 gene (954 bp) into the expression vector pET44b(+). The recombinant protein was expressed in Escherichia coli BL21(DE3) (Figure 4A) and purified using the Ni-Resin NTP column and eluted in 200 mM imidazole solution. Western blot analysis confirmed it was the mt-nd1 protein, as shown in Figure 4B.

The protein was immobilized on the Biacore NTA sensor chip. Seven different concentrations of Hup A (4.6625, 9.325, 18.75, 37.5, 150, 300, 600 µM) were used for the binding analysis. The KD value was determined to be about 4.961*10^−5^ (Figure 4C).

### SDS-PAGE analysis of Hup-MPs bound mouse brain tissue extracted proteins

**Figure 5:**
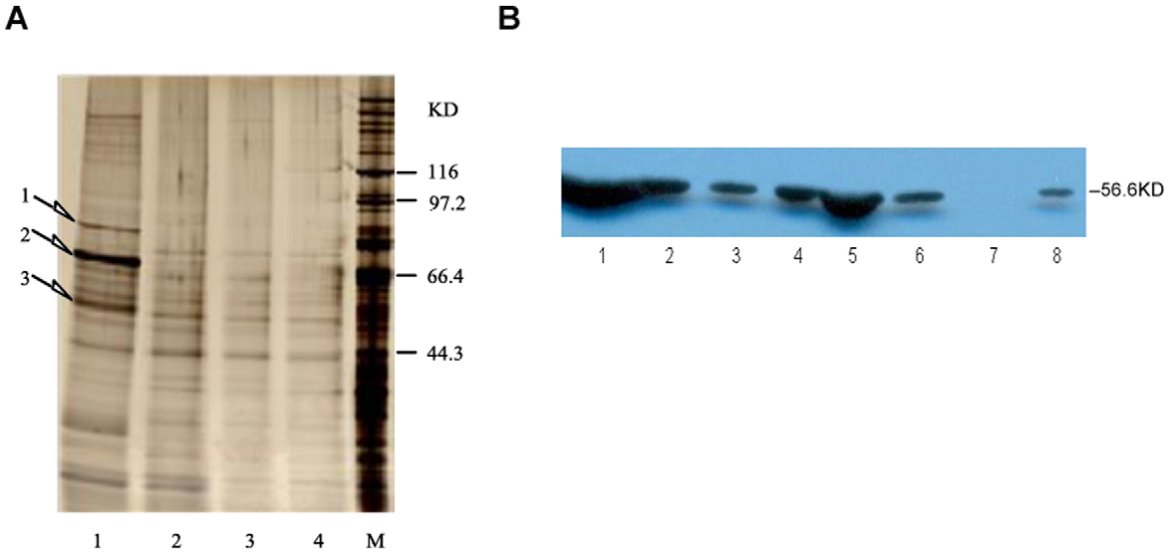
The possible protein-binding partners of Hup A selected from tissue lysate. (a)SDS-PAGE silver staining of the screen results of the possible protein-binding partners of Hup A. 1, Hup A elution solution of positive beads; 2, Hup A elution solution of control beads; 3, positive beads themselves; 4, control beads themselves; 5, Marker. (b)Western blot analysis of the drug-mitochondria ATP synthase possible interaction. 1,mitochondria lysate after interaction with positive beads; 2, 2nd time washing solution of positive beads; 3,3rd time washing solution of positive beads;4, positive beads themselves; 5, mitochondria lysate after interaction with negative beads; 6, 2nd time washing solution of negative beads; 7,3rd time washing solution of negative beads;8, negative beads themselves.

We also employed a similar strategy as plotted in Figure 2C to examine the interaction between Hup-MPs (or empty MP controls) and protein solution extracted from mouse brain tissue. Figure 5A showed the SDS-PAGE pattern of the proteins that were bound to Hup-MPs or empty MPs and then competed off by free Hup A. Lane 1 were the proteins bound to Hup-MPs, and lane 2 were the proteins bound to MPs. By comparing these two samples, we identified three major bands (at about 80 KD, 70 KD and 50 KD), which were observed reproducibly in repeated experiments. They were collected and sent out for mass spectrometry (MS) analysis. A few proteins were identified and listed in Table 3. Most notably there was the mitochondria ATP synthase which was also found in our cDNA phage library screening experiments. In order to further confirm the interaction between Hup A and mitochondria ATP synthase, we used a different Hup A conjugated MP using different linker structure the HupA-biotin-MPs. Ethonalamine-biotin-MPs were also made as the control. We isolated the mitochondria from mouse brain tissues and obtained the protein lysate. HupA-biotin-MPs were used to interact with the lysate and fish out the bound proteins. Figure 5B showed the western blot analysis of the bound protein eluted from HupA-biotin-MPs as compared to the control MPs. By comparing lane 3 and 7, lane 4 and 8, we confirmed that mitochondria ATP synthase did interact with HupA-biotin-MPs, but not or very weakly with the control MPs.

**Table 3:** Protein list identified by MS from the bands cut down from 4–12% SDS-PAGE silver staining gel.

Band number	Protein (IPI)	Protein description	Biological functions
**band 1**	IPI00329801.12	Annex in A5[Mus musculus]	an anticoagulant protein that acts as an indirect inhibitor of the thromboplastin-specific complex, which is involved in the blood coagulation cascade and also to inhibit the activity of phospholipase A1
**band 2**	IPI00345960.1	Ccdc55 Coiled-coil domain-containing protein 55	unknown
**band 3**	IPI00130280.1IPI00468481.2	Atp5a1 ATP synthase subunit alpha, mitochondrial precursorAtp5b ATP synthase subunit beta, mitochondrial precursor	catalyzing ATP synthesis using an electrochemical gradient of protons across the inner membrane during oxidative phosphorylation

## Discussion

Bioactive molecules identified from natural products have been an important source for drug discovery [20]. However in many cases their targets and molecular mechanisms were not fully understood. Furthermore, there have been many studies suggesting the multi-target effects of natural products [21], especially those derived from traditional Chinese medicine [22]. Therefore it is important and necessary to discover as many molecular mechanisms involved as possible. One way to investigate the drug's molecular mechanism is based on phenotype or pharmacological studies [12]–[14]. Another approach is to fish and identify the molecular target of the compound [15]–[17]. There have been studies developing various ‘fishing’ techniques such as drug affinity pull-down [23], drug affinity responsive target stability (DARTS) [18] and proteomics based affinity enrichment [24]. We described in this study a different approach employing the magnetic biopanning scheme (Figure 2), which is pretty straightforward and fast. Maekawa N, et al. used a similar approach to purify 15d-PGJ_2_'s interacting factors from crude cell extracts [25]. Under this scheme, the magnetic particles were suspended in the reaction solution, which would allow more complete interaction with the reactants. After the interaction, the magnetic particles can be easily pulled out using an outside magnetic field. But there is a possibility that the chemical conjugation may affect the binding characteristics of the compound [26]. Therefore it is very important to include binding experiments without the MPs to confirm the interaction between the drug itself and the possible targets (Figure 3 and 4).

In this study, we used two different biological systems for the biopanning. One is based on the cDNA phage display library. The proteins were displayed on the surface of the phages in many copies and have been widely used in binding studies [27]–[29]. The cDNA gene fragments were expressed and the displayed peptides may contain some binding domains in the natural protein. More importantly, the phage clones that were identified from the binding studies can be easily amplified for further panning studies and sequenced for target identification. The other system we used is the total proteins from brain tissue extracts. Since Hup A was thought to act upon targets in the brain mostly, we took the whole mouse brain tissue and isolated all the proteins. The bound proteins were characterized using Mass Spectroscopy.

Unfortunately, the protein candidates selected from the two systems had limited overlap, and the known target of Hup A, acetylcholinesterase (AChE), did not appear in the candidates list. There might be various reasons. For the cDNA phage screening, it might be that the cDNA display library didn't contain the AChE interactive domain gene. For the tissue lysate study, we think there is a possibility that the amine group on Hup A that we used to link to the magnetic particles was critical for binding with AChE. Although most studies had suggested the lactam ring and ethylidene methyl [26] on Hup A were main binding moieties, an earlier study had suggested that there were interactions between the amine group of Hup A with the aromatic groups of Trp 84 and Phe 330 of AChE and also ionic interactions between the amine group with the carboxyl groups of Asp 72 and Glu 199 of AChE [30]. We had tried to look for bindings between Hup-MPs with pure AChE purchased from Sigma. The interaction was not significant based on SDS-PAGE analysis. The Hup-MPs conjugation may have interfered with the binding between Hup A and AChE, and our screening method may be biased towards target bindings other than AChE. So the site of chemical conjugation on the drugs would have to be evaluated individually and carefully. In a similar effort to identify binding proteins of diadenosine tetraphosphate, we did find the known target, GroEL protein [31].

By comparing the results from both screening assays, we identified the mitochondria ATP synthase, which was highly enriched in one of phage screening efforts (Table 1) and also found in bound fraction of proteins from eukaryote tissue lysates (Figure 5). This is significant and there is a good chance that it could be a real target of Hup A.

Another possible protein target that was repeatedly found from the phage library was the mitochondria NADH dehydrogenase subunit 1. Interestingly it is also from the mitochondria and related to the ATP production in the respiratory chain. We not only confirmed the binding between that specific phage clone and Hup A using capillary electrophoresis (Figure 3), but also characterized the binding characteristics of the expressed protein and Hup A using SPR (Figure 4C). The binding constant calculated was 4.961*10^−5^, which is much higher than the binding constant between Hup A and AChE [32]. However, recent pharmacological studies had indeed pointed out the involvement of NADH dehydrogenase, also called complex I, in Hup A's activities [33], [34]. Gao X, et al reported that pretreatment with a certain concentration of Hup A could help to maintain NADH dehydrogenase activity and also promote ATP production when mitochondria was exposed to Aβ. So our data would support such an activity and even provide some underlying molecular mechanisms.

There have been numerous studies exploring the mechanisms of Hup A's activity in vivo. In addition to be a potent inhibitor of AChE [3], studies had suggested Hup A may also exert its neuroprotective effects through many pathways, such as antioxidation [35]–[37], antiapoptosis [4]–[6], attenuating the metabolism of the amyloid precursor protein(APP) [8], [9], protecting Ischemia Injury [10]. Based on our data and also some earlier pharmacological studies [33], [34], we think there should be some interaction between Hup A and mitochondria proteins. Since mitochondria is an important organelle that's involved in cell apoptosis [38], and one of the most important pathologies for AD is neuron apoptosis [39]–[41]. There was suggestions of relationships between mitochondria dysfunction and AD [42]–[47]. Hup A may affect mitochondrial functions and help to prevent neuron apoptosis for the treatment of AD and other neurodegenerative diseases. Specifically, we think Hup A may interact with mitochondria main matrix enzymes including NADH dehydrogenase and ATP synthase, which may coexist as lipid raft complexes [48], and improve their activities, promote electron transport on the respiratory chain, and increase ATP production to supply energy for neuronal repair. Such a molecular mechanism agrees well with most published observations.

Furthermore, since we have identified quite a few possible targets, especially the ones in italic as listed in Table 1 and 3. It may be useful to look at the whole repertoire of selected possible targets, which may include some naturally less abundant or unstable proteins that could not be picked up using conventional methods. Although the types of proteins and their abundance in the two different systems were different, the proteins most likely to be picked up were quite different. We only selected two candidates as possible targets and carried out more binding validation studies. The purposes of the study were not only trying to find the various possible molecular targets of Hup A, but also to demonstrate the feasibility of using magnetic biopanning as a viable method for investigating the complex molecular mechanisms of bioactive molecules. The detailed scheme would need to be optimized further, including chemical conjugation site selection and binding assay conditions. But such a method has potentials in the identification of bioactive molecule-protein interaction, and will probably contribute more in future interactome study of small molecules.

## Materials and Methods

### Ethics Statement

Mice were housed in the specific pathogen-free Animal Centre of Shanghai Jiao Tong University. All experimental procedures were approved by the Animal Experimental License Number of SYXK 2007-0025-0125 and done according to The Animal Care & Welfare Committee of Shanghai Jiao Tong University.

### Materials

Huperzine A, 1,1′-Carbonyldiimidazole N,N′ (CDI), 18-Crown -6 were purchased from Sigma-Aldrich. BrMmC 4-bromomethyl- 7-methoxycoumarin was bought from ACROS ORGANICS (New Jersey USA).T7 Select®10-3b phage cDNA library and pET44b(+)vector were purchased from Novagen. Human liver cDNA library was purchased from Beijing BioEev-Tech. Scientific&Technical Co.,Ltd. RIPA lysis buffer was purchased from SANTA CRUZ. Protease Inhibitors were purchased from Merck. Ni-NTA Fast Start Kit was purchased from Qiagen. C57BL/6 mice were bought from Shanghai SLAC laboratory animal Co.Ltd. MT-ND1 antibody(ab7425) was purchased from Abcam. DyLight 680 Goat Anti-Rabbit IgG was purchased from KPL. EZ-Link Sulfo NHS-SS Biotinylation Kit was purchased from Thermo scientific. Mitochondria protein isolation kit was purchased from Sangon Biotech(Shanghai)Co., Ltd.. ATP synthase subunit βmonoclonal antibody(A21351) and Dynabeads M-280 Streptavidin(112.05D) were purchased from Invitrogen. Goat anti-mouse IgG(H+L) HRP(GAM007)was purchased from Multisciences Biotech Co., LTD.. All other chemicals used were of analytical grade.

### Preparation of Hup-MPs

Magnetic nanoparticles were synthesized by co-precipitation method in the presence of a polymer matrix as described By Liu et al [19]. The nanoparticle-polymer matrix was then stabilized by crosslinking the carboxyl groups on the polymer backbone using small amount of CDI for 2 hours in acetone at RT and the reaction was stopped with ethylenediamine. The resulted magnetic particles (MP) can then be collected by applying a magnetic field. They were washed 3 times by ddH_2_O and lyophilized for future use.

Hup A were conjugated to the MPs by dissolving the MPs in acetone and activated with excess amount of CDI. The activated MPs were collected by magnetic separation, dissolved in PBS, and mixed with Hup A in THF. They were reacted for 2 hours, and the finally got Hup-MPs were collected by magnetic separation, washed 3 times by PBS, and lyophilized.

### FTIR analysis of the got Hup-MPs

The Hup-MPs particles were analyzed by FTIR spectroscopy(Paragon 1000, Perkin Elmer,USA).

### Hup-MPs mediated interaction and selection from phage and protein libraries

The Hup-MPs and MPs(MPs without Hup A were used as control) were respectively dissolved in PBS, blocked with BSA overnight, and washed 3 times by PBS. They were then incubated with the solution containing either the T7 phage cDNA library or tissue extracts for 2 hours at 4°C. The phage or protein bound Hup-MPs or MPs were collected by magnetic separation and washed using 0.1% Tween 20 in PBS 7 times. (In the phage screening study, 5 consecutive screenings were done and the washing buffers were changed to 0.2%, 0.3%, 0.4%, 0.5% Tween 20 concentration sequentially). The bound phages or proteins were finally harvested by adding collected Hup-MPs or MPs to Hup A solution and dissociation from the Hup-MPs or MPs by competition.

### Phage library preparation, amplification and analysis

The T7 phage library was obtained from Novagen and amplified according to the protocol supplied. The selected phages obtained after interacting with HupA-MPs were amplified by infecting E. coli BL21(5403) and purified for the next round of screening. After the screening effort, the selected phage clones competed down by dissociative Hup A were collected and the cDNA inserts were amplified by PCR (upstream primer:5′- ggagctgtcgtattccagtc- 3′ and downstream primer:5′- aacccctcaagacccgttta - 3′) and analyzed by gene sequencing. The gene sequences obtained were blasted in the NCBI nr database to identify the target genes.

### Capillary electrophoresis analysis of phage drug interaction

The phages were purified through CsCl density gradient centrifugation and dialyzed in PBS for 3 times. Capillary zone electrophoresis experiments were done using the P/ACE TM MDQ (Beckman Coulter) apparatus. The capillary was first filled with Hup A containing buffer solution (Na_2_HPO_4_ 40 mM, NaH_2_PO_4_ 40 mM, pH7.5) with fixed drug concentration (Df). Then different mixtures of drug and phage at various ratios were injected and analyzed. The drug concentrations in the mixtures were 0.0001 mg/ml, 0.0002 mg/ml, 0.0004 mg/ml, 0.001 mg/ml and 0.002 mg/ml while the amount of phages was constant. The capillary electrophoresis conditions were 4.0 s injection time, 0.5 psi injection pressure, 6 KV separating voltage, 30 min separating time, 214 nm UV testing wavelength. Hummel-dreyer method was used to calculate the concentration of Hup A combined with phages Db [49]–[51],which is equal to the difference between the injected drug concentration and Df when the minus peak of drug disappeared.

### Expression and purification of MT-ND1

The mitochondrion NADH dehydrogenase subunit 1 gene was cloned from human liver cDNA library by specific primers (upstream: 5′- tcccccggggcatacccatggccaac - 3′, and downstream: 5′- ccgctcgagggtttgagggggaatgct-3′) and incorporated into the SmalΙ and XholΙ sites of pET44b(+)(Novagen) expression vector, which was then transformed into E. coli strain BL21(DE3)(Chemically Competent cells from Invitrogen). To obtain MT-ND1, the transformed BL21(DE3) was cultured in the presence of 1 mM IPTG for 20 hours at 28°C. After extraction, MT-ND1 were harvested from E. coli supernatant by passing through Ni - NTA column, and being eluted with 5 mM and 200 mM imidazole solution (pH 7.0) respectively. The eluted proteins were further purified by ultracentrifugation and dialyzed against PBS buffer (pH 7.4).

### Western-blot analysis of the expressed MT-ND1

The expressed protein after purification was detected by western blot analysis. Briefly, the samples with different amount of protein were mixed with loading buffer, boiled for 5 minutes, separated by 4–12% Bis-Tris gel through SDS-PAGE and electrotransferred to a nitrocellulose(NC) membrane with the iBlot® Dry Blotting System (Invitrogen). The NC membrane was subsequently blocked with blocking buffer at room temperature for 2 hours, washed 3 times by TBST with 0.05% tween-20, and incubated at 4°C for 12 h with monoclonal rabbit anti-human MT-ND1 antibody. After being washed 3 times, the membrane was labeled with DyLight 680 Goat Anti-Rabbit IgG for 1 hour at room temperature, washed 3 times again, and finally photographed by LI-COR Odyssey* Infrared Imaging System.

### Surface plasmon resonance (SPR) binding assay of the interaction between Hup A and MT-ND1

SPR Binding analysis between Hup A and MT-ND1 was carried out on a Biacore ×100 instrument (GE healthcare, USA) using a Nitrilotriacetic acid (NTA) Chip (GE healthcare, USA) [52], [53]. The NTA chip was first activated with nickel ions by passing 500 uM NiCl_2_ in NTA running buffer (10 mM HEPES, 150 mM NaCl, 50 uM EDTA, 0.005% v/v surfactant P-20, pH 7.4) as instructed. Secondly, the purified proteins, after being confirmed MT-ND1 by western blot assay were introduced for 480 seconds to allow the protein to be immobilized on the chip surface. The surface was then washed using NTA running buffer containing 3.5 mM EDTA for 240 seconds. Subsequently, drug solutions with concentrations ranging from 4.6625 uM to 600 uM were sequentially introduced to flow through the MT-ND1 immobilized surface for 180 seconds each. The binding data was processed and the steady state affinity calculation was carried out using the Biacore ×100 Evaluation Software (GE healthcare, USA). The chip surface was regenerated using 0.35 M EDTA, pH 8.3 at the end of each cycle.

### SDS-PAGE analysis of tissue lysate

Mouse brain tissue was collected from 2 healthy C57BL/6 mice (16 weeks old) after they were anesthetized by lethal injection of ketamine and then were perfused intracardially with 25 ml of normal saline. Brain tissues were rapidly put in ice cold RIPA lysis buffer (containing protease inhibitor cocktail, pmsf and sodium orthonavate), and homogenized, and centrifuged at 12,000 rpm for 10 mins at 4°C to collect the supernatant.

The tissue lysate before and after interaction with Hup-MPs were examined by 4–12% SDS-PAGE, operated according to the protocol of NuPAGE® Novex 4–12% Bis-Tris gel from Invitrogen. SilverQuest Silver Staining Kit was used to stain the gel after electrophoresis.

### Mass spectrometry identification of specific proteins

3 specific protein bands were fished out repeatedly in the 2 screen experiments and were cut down and sent to Shanghai Applied Protein Technology Co., Ltd. for MS identification using the LTQ, Thermo Finnigan apparatus. The analysis parameters were as following: positive charge testing mode, micro spray injecting way, 170°C capillary temperature, 0.15 mm*15 cm column, 400–2000 DAL scanning scope.

### Western blot assay of Hup A and mitochondria ATP synthase binding

The binding between Hup A and mitochondria ATP synthase was validated by western-blot analysis. Mitochondria was isolated from mouse brain tissue using Mitochondria protein isolation kit purchased from Sangon Biotech (Shanghai) Co., Ltd., and lyzed in the specific buffer (20 mmol/L Tris-HCI,pH7.5, 2 mmol/L EGTA, 2 mmol/L EDTA,1% Triton X-100, 1 ul/ml protease inhibitor and DTT) on ice for 100 minutes. The supernatant after centrifugation was dialyzed in PBS overnight at 4°C for further use. 5 mg Hup A and 150 ul 50 mM ethanolamine (as control) was biotinylated with 2 mg EZ-Link Sulfo-NHS-SS-Biotin according to the kit's protocol. Biotin-Hup A and biotin- ethanolamine were then loaded on the surface of 200 ul Dynabeads M-280 with streptavidin. Half of the beads with Hup A or ethanolamine were allowed to interact with 100 ul of the mitochondria lysate at 700 rpm, 4°C for 3 hours, and then washed 3 times by 500 ul PBS each time. The samples from the positive beads (beads with Hup A) and the negative beads(beads with ethanolamine), the supernatant after interacting with beads, the washing buffer, and the beads themselves were lyophilized and resuspended in 50, 30 and 25 ul ddH_2_O, loaded on 10% SDS-PAGE. The target protein, mitochondria ATP synthase, was identified using ATP synthase subunit β monoclonal antibody from mouse (A21351 from Invitrogen) and stained with goat anti-mouse IgG(H+L) HRP.
